# Trans-catheter aortic valve implantation through the ascending aorta in a patient with previous right pneumonectomy

**DOI:** 10.1186/1749-8090-8-S1-P178

**Published:** 2013-09-11

**Authors:** M Fernández, JR Echevarría, G Laguna, H Valenzuela, P Pareja, M Blanco

**Affiliations:** 1 H. Clínico Universitario de Valladolid, Spain

## Background

We report a successful trans-catheter aortic valve implantation using a right-sided thoracotomy in a patient with right pneumonectomy not suitable for conventional surgery, trans-femoral or transapical approach.

## Methods

A 74 year old male with severe aortic stenosis was referred for consideration of aortic valve replacement. He was highly symptomatic with both angina and dyspnea (NYHA class III). The echocardiogram confirmed moderate depression of LVEF. Coronary angiography demonstrated severe stenosis of the left-descending and right coronary arteries. His comorbidities included diabetes mellitus, peripheral vascular disease and left pneumonectomy, with residual poor lung function. His logistic EuroSCORE was 16.6%. Two drug eluting stents were implanted. Trans-femoral route was not feasible because of a small-caliber femoral arteries and severe peripheral vasculopathy, including subclavian severe stenosis. Trans-apical approach was also not desirable because the ventricular apex was shifted to the right and would have posed technical.

Upper partial thoracotomy 5 cm far from the right border of the sternum at the second intercostal space and partial resection of the rib was performed and the ascending aorta was exposed. An 18-Fr sheath was introduced through a small puncture 6 cm over the aortic plane. Valvulotomy was performed with a 25-mm balloon and a 29-mm device was then implanted.

## Results

A good result was obtained with minimal paravalvular leak. He did not developed heart failure or auriculo-ventricular block, but sub-acute right-sided limb ischemia appeared 72 hours from the procedure, which required percutaneous revascularization. Within one year of follow-up he remains asymptomatic from a cardiovascular point of view.

## Conclusions

This is our first experience of an aortic prosthesis implanted using right-side thoracotomy in a patient not suitable for other approaches, technique that can be used in the future in selected patients.

